# Correlation between Plasma Macrophage Migration Inhibitory Factor Levels and Long-Term Prognosis in Patients with Acute Myocardial Infarction Complicated with Diabetes

**DOI:** 10.1155/2019/8276180

**Published:** 2019-03-07

**Authors:** Haiyi Yu, Xinyu Wang, Xiangning Deng, Youyi Zhang, Wei Gao

**Affiliations:** ^1^Department of Cardiology and Institute of Vascular Medicine, Peking University Third Hospital, Beijing, China; ^2^NHC Key Laboratory of Cardiovascular Molecular Biology and Regulatory Peptides, Beijing, China; ^3^Key Laboratory of Molecular Cardiovascular Science, Ministry of Education, Beijing, China; ^4^Beijing Key Laboratory of Cardiovascular Receptors Research, Beijing, China

## Abstract

Macrophage migration inhibitory factor (MIF), a widely expressed pleiotropic cytokine, is reportedly involved in several cardiovascular diseases, in addition to inflammatory diseases. Plasma MIF levels are elevated in the early phase of acute cardiac infarction. This study is aimed at investigating the correlation between plasma MIF levels and cardiac function and prognosis in patients with acute ST-segment elevation myocardial infarction (STEMI) with or without diabetes mellitus. Overall, 204 patients with STEMI who underwent emergency percutaneous coronary intervention were enrolled: 57 and 147 patients in the diabetes and nondiabetes STEMI groups, respectively. Sixty-five healthy people were selected as controls. Plasma MIF levels were measured at the time of diagnosis. Basic clinical data and echocardiographic findings within 72 h of admission were collected. Patients were followed up, and echocardiograms were reviewed at the 12-month follow-up. Plasma MIF levels were significantly higher in the diabetes and nondiabetes STEMI groups than in the control group and in patients with Killip grade ≥ II STEMI than in those with Killip grade I. Plasma MIF levels were negatively correlated with the left ventricular ejection fraction (LVEF) of myocardial infarction in patients with or without diabetes in the acute phase of infarction, whereas the left ventricular diastolic dysfunction (LVDD) was positively correlated. MIF levels in the nondiabetes STEMI group were positively correlated with N-terminal pro-b-type natriuretic peptide levels and were associated with LVEF and LVDD at the 12-month follow-up. The risk of adverse cardiovascular and cerebrovascular events was significantly higher in the MIF high-level group (≥52.7 ng/mL) than in the nondiabetes STEMI group 36 months after presentation. Thus, MIF levels in STEMI patients with or without diabetes can reflect acute cardiac function. In STEMI patients without diabetes, MIF levels can also indicate cardiac function and long-term prognosis at the 12-month follow-up.

## 1. Introduction

Acute myocardial infarction is a clinically critical disease with increasing incidence, and its long-term prognosis is significantly associated with infarction-induced heart failure [1]. In recent years, acute and long-term outcomes have significantly improved with the development of coronary interventions, aggressive anticoagulation, and antiplatelet therapy. However, there have still been higher recurrent major adverse cardiovascular events in some patient populations, especially acute myocardial infarction in type 2 diabetes mellitus patients. Diabetes mellitus is associated with a markedly increased risk for cardiovascular diseases and death, which was univocally confirmed by results from the Whitehall study [2]. Identifying biomarkers that are elevated in early-stage acute myocardial infarction complicated with diabetes and that have a certain suggestive effect on cardiac function after infarction is necessary.

Macrophage migration inhibitory factor (MIF), a pleiotropic protein with inflammatory chemokine activity, is involved in chronic inflammatory processes, such as atherosclerosis [3]. Circulating MIF levels increase early in patients with myocardial infarction and can reflect the myocardial infarct size [3]; however, the relationship between MIF levels and acute and chronic cardiac function after infarction remains unclear. This study is aimed at investigating the effect of diabetes mellitus on plasma MIF levels in the early stage of the disease in patients with acute ST-segment elevation myocardial infarction (STEMI) and analyzing the relationship between MIF levels and cardiac function indicators and long-term prognosis after myocardial infarction.

## 2. Materials and Methods

### 2.1. Ethical Approval

The study was conducted according to the Declaration of Helsinki and was approved by the Ethics Committee of Peking University Third Hospital. Informed written consent was obtained from all participants before their enrollment.

### 2.2. Study Design and Population

From September 2011 to March 2013, 204 patients who both met the 2009 American College of Cardiology (ACC)/American Heart Association (AHA) acute STEMI diagnostic criteria and were admitted to Peking University Third Hospital were included. The inclusion criteria were as follows: (1) patients are older than 18 years, (2) onset of acute myocardial infarction symptoms to visit time was <12 h, and (3) patients had undergone emergency coronary angiography and percutaneous coronary intervention (PCI). Patients with acute coronary syndrome or related symptoms in the past month, valvular heart disease, cardiomyopathy, coinfection status, malignant tumor, autoimmune disease, blood disease, and severe liver and kidney dysfunction and/or treatment with antibiotics, steroid hormones, immunosuppressants, or other anti-inflammatory drugs were excluded. During the same study period, 65 healthy age- and sex-matched volunteers were selected as controls (control group).

The patients were divided into a nondiabetes STEMI group (147 cases, no prediabetes history and admission glycosylated hemoglobin (HbA1c) < 6.5%) and a diabetes STEMI group (57 cases) according to the history of diabetes mellitus and HbA1c. The patients in the nondiabetes STEMI group were further divided into those with stress-induced hyperglycemia (*n* = 31; fasting blood glucose level ≥ 7.0 mmol/L or random blood glucose level ≥ 11.1 mmol/L) and those with normal blood glucose status (*n* = 116).

### 2.3. Specimen Collection and Storage

For plasma analysis, 4 mL of venous blood was collected from all 204 patients and 65 healthy participants in the control group early in the morning, after fasting, in an ethylenediaminetetraacetic acid (EDTA) anticoagulant tube, immediately placed in an ice box, and transported at 4°C within 30 min after collection to the central laboratory. It was then centrifuged at 1000 × g for 15 min at 4°C within 6 h after collection, and the upper layer of the plasma was collected. Aliquots of the EDTA plasma were numbered and stored in a freezer at −80°C until analysis; repeated freeze-thaw cycles were avoided.

### 2.4. Assays

Plasma MIF levels were measured using an enzyme-linked immunosorbent assay kit (R&D, USA) according to the manufacturer's instructions, with a detection range of 156 pg/mL-10000 pg/mL and a minimal detection limit of 25 pg/mL. All healthy controls and STEMI patients had detectable levels of circulating MIF in this study. STEMI patients were tested for routine blood and random blood glucose at the time of presentation. The peak values of high-sensitive troponin T (hs-TnT) were determined by blood detection before and every 6 h after PCI up to 48 h. Fasting blood glucose, HbA1c, N-terminal pro-b-type natriuretic peptide (NT-pro-BNP), and hypersensitive C-reactive protein (hs-CRP) levels were detected within 24 h after onset. Routine blood examination was performed using an automated hematology analyzer (XE2100, Sysmex, Kobe, Japan). Hs-TnT and Nt-pro-BNP levels were detected using an E601 immunoassay analyzer (Roche Diagnostics, Mannheim, Germany). The remaining biochemical indicators were detected using the AU5400 automatic chemical analyzer (Beckman Coulter, California, USA). Estimated glomerular filtration rates (eGFR) were calculated according to the Cockcroft-Gault formula.

### 2.5. Assessment of Coronary Angiography and Echocardiography Examination

STEMI patients underwent emergency coronary angiography and PCI. Coronary lumen diameter reduction ≥ 50% was considered clinically significant lesions. The Gensini score was used to assess the severity of coronary lesions [4]. Echocardiography was completed within 72 h of admission. The left ventricular end-diastolic and end-systolic volumes were measured using the dual-plane modified Simpson method using the Vivid 7 instrument (General Electric Company, Connecticut, USA), and the left ventricular ejection fraction (LVEF) was also calculated. One year after myocardial infarction, echocardiography was performed for patients who agreed to visit the hospital for reexamination.

### 2.6. Follow-Up

All STEMI patients were followed up through routine outpatient or telephone follow-up. The follow-up period was 36 months. Major adverse cardiovascular and cerebral events (MACCEs) were recorded at 1, 3, and 6 months after discharge and every 6 months thereafter. MACCE includes all-cause death, cardiac death, nonfatal myocardial reinfarction, revascularization, rehospitalization owing to heart failure, and stroke. The follow-up was discontinued when the first endpoint occurred.

### 2.7. Statistical Analyses

Statistical analysis was performed using GraphPad Prism software (version 5.0, GraphPad Software Inc., San Diego, California, USA). Continuous variables were expressed as mean ± SD. Analysis of variance was used to compare the results of the three groups. Categorical variables were expressed as percentage or rate, and Pearson's *χ*^2^ test was used to compare results among the groups. Nonnormally distributed data were expressed as the median (quartile) [*M*(Q25, Q75)], and the Mann-Whitney rank sum test was used to compare MIF levels between two groups. Correlation analysis was performed using the Spearman correlation test, and the Kaplan-Meier survival analysis curve was used to evaluate the predictive value of MIF for prognosis. A *P* value < 0.05 was considered statistically significant.

## 3. Results

### 3.1. Baseline Characteristics of Study Participants

White blood cell counts and hs-CRP and low-density lipopolysaccharide cholesterol (LDL-c) levels were significantly higher in the diabetes and nondiabetes STEMI groups than in the control group (both *P* < 0.05, Table 1). Among patients in the diabetes STEMI group, the average age was 60.6 ± 11.5 years, 44 were men (77.2%), 47 cases (82.5%) presented with onset to visit time < 6 h, and 22 cases presented with anterior myocardial infarction (38.6%). There was no significant difference in the above conditions between the nondiabetes and diabetes STEMI groups (all *P* > 0.05, Table 1). The incidence of hypertension and Killip grade ≥ II was significantly higher in the diabetes STEMI group than in the nondiabetes STEMI group (both *P* < 0.05, Table 1). Coronary angiography indicated that the proportion of patients with multivessel disease was higher in the diabetes STEMI group than in the nondiabetes STEMI group (45.6% vs. 32.0%, *P* = 0.049). Random blood glucose and HbAlc (glycosylated hemoglobin) levels and eGFR were significantly lower in the nondiabetes STEMI group than in the diabetes STEMI group (all *P* < 0.05, Table 1); other clinical or biochemical factors, such as Nt-pro-BNP, TnT peak, hs-CRP, and blood lipid, were comparable between the two groups (all *P* > 0.05, Table 1).

### 3.2. Comparison of Plasma MIF Levels in Different Populations

**Figure 1:**
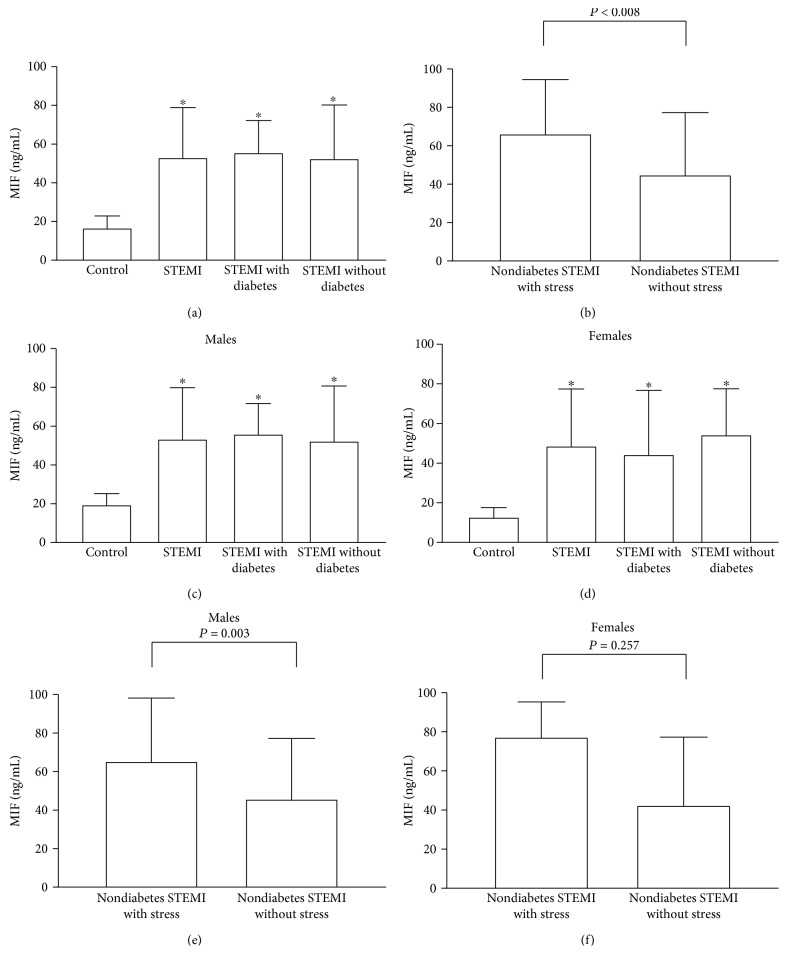
Comparison of plasma macrophage migration inhibitory factor (MIF) levels in different populations. (a) MIF concentrations were evaluated in all controls (*n* = 65) and ST-segment elevation myocardial infarction (STEMI) with (*n* = 57) and without (*n* = 147) diabetes. (b) In the nondiabetic STEMI group, plasma MIF levels between the stress-induced hyperglycemia group (*n* = 31) and the group with euglycemia (*n* = 116) were significantly different. Stratified according to sex and disease type, the graph shows the plasma MIF levels of patients with STEMI compared to those of the healthy control group, including STEMI with and without diabetes in males (c) and females (d), respectively. The plasma MIF levels of nondiabetic STEMI male patients (e) between the stress-induced hyperglycemia group and the group with euglycemia were significantly different, but there was no significant difference in those of females (f). ^∗^*P* < 0.05.

Plasma MIF levels were significantly elevated in patients with STEMI compared to those in the healthy control individuals (53.1 (36.4-81.9) pg/mL vs. 16.9 (12.8-22.9) pg/mL, *P* < 0.001, Figure 1(a)). However, no significant difference in plasma MIF levels was observed between the nondiabetes and diabetes STEMI groups (55.8 (40.1-72.2) pg/mL, 52.7 (34.2-80.2) pg/mL, *P* = 0.683, Figure 1(a)). In the nondiabetes STEMI group, plasma MIF levels were significantly higher in the stress-induced hyperglycemia group than in the euglycemia group (66.3 (47.8-94.4) pg/mL vs. 45.0 (32.2-72.3) pg/mL, *P* = 0.008, Figure 1(b)).

Stratified according to sex and disease type, plasma MIF levels in patients with STEMI were significantly elevated compared to those in the healthy control subjects both in males (19.7 (14.7-25.3) pg/mL vs. 53.6 (37.9-79.9) pg/mL, *P* < 0.001, Figure 1(c)) and in females (13.0 (11.4-17.7) pg/mL vs. 48.86 (32.9-77.5) pg/mL, *P* < 0.001, Figure 1(d)). No significant difference in plasma MIF levels was observed between the nondiabetes and diabetes STEMI groups both in males (52.6 (36.7-80.7) pg/mL vs. 56.1 (40.7-71.7) pg/mL, *P* = 0.621, Figure 1(c)) and in females (44.6 (37.2-76.7) pg/mL vs. 54.6 (29.8-77.5) pg/mL, *P* = 0.815, Figure 1(d)). The plasma MIF levels of nondiabetic STEMI male patients were significantly different between the stress-induced hyperglycemia group and the group with euglycemia (66.3 (47.8-94.4) pg/mL vs. 45.0 (32.2-72.3) pg/mL, *P* = 0.008, Figure 1(e)), but there was no significant difference in those of females (77.4 (49.8-95.3) pg/mL vs. 42.7 (29.5-77.3) pg/mL, *P* = 0.257, Figure 1(f)).

### 3.3. Relationship between Plasma MIF Levels and Killip Grading and Nt-pro-BNP in the Diabetes STEMI and Nondiabetes STEMI Groups

Plasma MIF levels in patients were not correlated with age (*r* = 0.205, *r* = 0.067, both *P* > 0.05), eGFR (*r* = −0.020, *r* = −0.037, *P* > 0.05), or HbAlc (*r* = 0.122, *r* = 0.106, both *P* > 0.05) in either the diabetes STEMI or nondiabetes STEMI groups. However, plasma MIF levels were positively correlated with hs-TnT peak values in the diabetes and nondiabetes STEMI groups (*r* = 0.343 and *r* = 0.474, respectively, both *P* < 0.01). In addition, plasma MIF levels were positively correlated with random blood glucose and hs-CRP levels and Gensini scores in the nondiabetes STEMI group (*r* = 0.326, *r* = 0.186, and *r* = 0.301, all *P* < 0.05), but MIF levels in the diabetes STEMI group were not associated with them (*r* = 0.098, *r* = 0.194, and *r* = 0.161, all *P* > 0.05).

Plasma MIF levels were significantly higher in patients with Killip grade ≥ II STEMI than in those with Killip grade I (70.3 (45.5-93.5) vs. 50.5 (37.0-75.6), *P* = 0.039). There was no significant difference in MIF levels between patients with Killip grade ≥ II and those with Killip grade I (*P* > 0.05). MIF levels in the nondiabetes STEMI group were positively correlated with Nt-pro-BNP at 24-72 h after admission (*r* = 0.298, *P* < 0.001), whereas MIF levels in patients with diabetes STEMI were not associated with Nt-pro-BNP (*r* = 0.072, *P* = 0.595).

### 3.4. Relationship between Plasma MIF Levels in Patients with Diabetes and Nondiabetes STEMI and Cardiac Function in the Acute Phase and Postoperative 12 Months.

**Figure 2:**
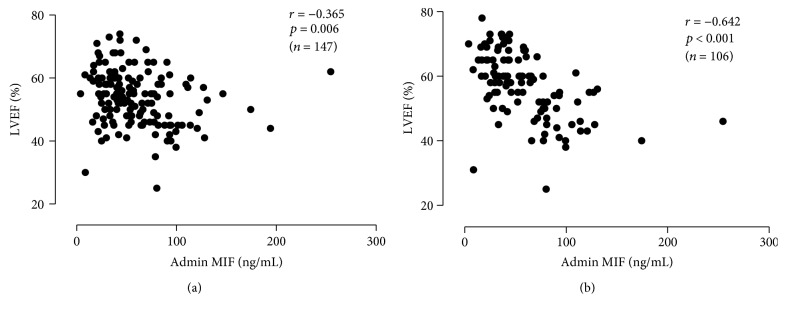
Correlation between plasma macrophage migration inhibitory factor (MIF) levels in different populations and cardiac function in the nondiabetes ST-segment elevation myocardial infarction (STEMI) group in the acute phase and postoperative 12 months. (a) Plasma MIF levels were correlated with left ventricular ejection fraction (LVEF) within 72 h of admission in the diabetic STEMI group. (b) MIF levels in the nondiabetic group were correlated with LVEF at the 12-month follow-up.

Plasma MIF levels of patients in the diabetes and nondiabetes STEMI groups were negatively correlated with LVEF within 72 h of admission (*r* = −0.336 and *r* = −0.365, respectively, both *P* < 0.01, Figure 2(a)) but were positively correlated with LVDD (*r* = 0.198, *r* = 0.301, all *P* < 0.05). MIF levels in the nondiabetes STEMI group were also associated with LVEF and LVDD at the 12-month follow-up (*r* = −0.642, *r* = 0.314, both *P* < 0.001, Figure 2(b)). However, blood MIF levels in the diabetes STEMI group were not associated with LVEF and LVDD (*r* = −0.257, *r* = −0.050, both *P* > 0.05).

### 3.5. Relationship between Plasma MIF Levels and Long-Term Prognosis in the Diabetes and Nondiabetes STEMI Groups

**Figure 3:**
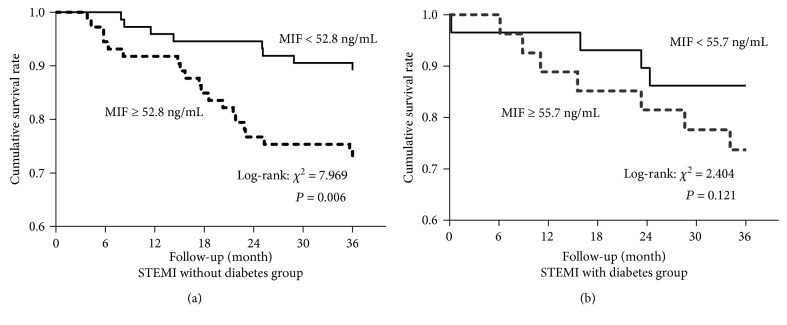
Survival analysis curve for STEMI patients grouped according to plasma macrophage migration inhibitory factor (MIF) levels. (a) Kaplan-Meier survival analysis shows a significantly increased risk of major adverse cardiovascular and cerebral events (MACCE) in the patients with nondiabetes ST-segment elevation myocardial infarction (STEMI) with plasma MIF ≥ 52.8 ng/mL at presentation; (b) in the diabetes STEMI group, the patients with MIF ≥ 55.7 ng/mL had no significant difference in the risk of MACCE compared to the patients with low MIF levels.

The patients were followed up for 36 months. During the follow-up period of the diabetes STEMI group, MACCE (22.8%) occurred in 13 patients, including three all-cause deaths, two cardiovascular deaths, four strokes, four heart failure hospitalizations, two myocardial infarctions, and two revascularizations. During the follow-up period of the nondiabetes STEMI group, MACCE (18.3%) occurred in 27 patients, including six all-cause deaths, four cardiovascular deaths, nine strokes, six patients with heart failure, three myocardial infarctions, and three people with revascularization. Kaplan-Meier survival analysis showed a significant increase in the risk of MACCE inpatients with nondiabetes STEMI having plasma MIF levels ≥ 52.8 ng/mL at presentation (*P* = 0.006, Figure 3(a)); in the diabetes STEMI group, patients with MIF levels ≥ 55.7 ng/mL had no significant difference in the risk of MACCE compared to those with low MIF levels (*P* = 0.121, Figure 3(b)).

## 4. Discussion

Currently, many studies have observed that blood MIF levels in patients with STEMI are elevated in the early stage [3, 5–7] but its relationship with cardiac function is rarely reported. In view of the previous suggestion that plasma MIF levels are significantly higher in patients with type 2 diabetes than in healthy people [8, 9], we further compared plasma MIF levels in STEMI patients with or without diabetes mellitus. Plasma MIF levels in the diabetes and nondiabetes STEMI group were positively correlated with the extent of acute cardiac function impairment, including Killip grade ≥ II, LVEF, and LVDD acute phase. Furthermore, MIF levels in nondiabetes STEMI patients at presentation were associated with Nt-pro-BNP levels in the acute phase and LVEF and LVDD at the 12-month follow-up and MIF levels had a predictive value for the occurrence of long-term cardiovascular and cerebrovascular adverse events.

As a proinflammatory factor, MIF is expressed in cells or tissues such as inflammatory cells, cardiomyocytes, islet cells, hypothalamus, and blood vessels [10]. MIF plays an important role in pathophysiological processes such as atherosclerosis, diabetes, unstable plaque formation, and stress reaction [11–14]. MIF is also an immunoregulatory cytokine, playing an important role in autoimmune and inflammation-related diseases, including inflammatory bowel diseases (IBD), systemic lupus erythematosus (SLE), rheumatoid arthritis (RA), type 1 diabetes mellitus, and multiple sclerosis (MS) [15]. In the current study, to focus on evaluating the relationship between MIF and cardiac function impairment in STEMI patients, we excluded patients with autoimmune diseases, which may affect MIF concentrations dramatically.

In this study, plasma MIF levels were significantly higher in STEMI patients with and without diabetes than in healthy control individuals and nondiabetes STEMI patients with stress hyperglycemia had higher MIF levels than those with euglycemia. It was reported that patients with coronary heart disease and diabetes have higher blood MIF levels than nondiabetes patients with coronary heart disease [8]. However, we did not observe these differences between diabetes and nondiabetes STEMI patients in our study, which may be related to the selection of patients with acute myocardial infarction as subjects. The source of elevated MIF levels in patients with myocardial infarction was mainly necrotic cardiomyocytes [10]. The sharp increase in MIF levels in the circulating blood after myocardial infarction may conceal the increased MIF levels caused by diabetes.

It is worth noting that this study found that MIF levels were significantly higher in nondiabetes STEMI patients with stress hyperglycemia than in those with euglycemia, suggesting that MIF is related to stress response. Among the nondiabetes STEMI patients, plasma MIF levels of the stress-induced hyperglycemia group and the group with euglycemia were significantly different in male patients but there was no significant difference in those in females. These results are consistent with a previous study, which reported that stress could elevate MIF levels in patients [14]. Further, these results suggest that MIF has a sex-specific effect, as was reported in a MS disease study [16, 17]. We found no difference in MIF levels between male and female patients or male and female healthy controls, but different levels of plasma MIF between the stress-induced hyperglycemia and the euglycemia groups were only found in male patients, suggesting that the pathogenetic effects of MIF in nondiabetes STEMI are sex dependent.

We found that plasma MIF levels in patients with diabetes mellitus and nondiabetes STEMI could reflect the extent of impaired cardiac function in the acute phase, probably because MIF levels in the early stage of infarction could reflect the myocardial infarct size indicated by myocardial zymogram changes and imaging [3, 7] and the size of myocardial infarction is one of the main factors that affected acute cardiac function impairment after myocardial infarction. Animal experiments demonstrated that the main source of MIF in the early phase after myocardial infarction is necrotic cardiomyocytes and its secretion pattern is characterized by direct, massive, and rapid release under ischemic stimulation, which is not dependent on de novo synthesis [10, 18]. Fan et al. [19] found that the MIF gene expression levels in cardiomyocytes were 30 times higher than that in skeletal muscle cells. Chan et al. [3] established a mouse coronary occlusion model and found that the MIF content in the damaged myocardium decreased by approximately 40%, and a loss of MIF in the damaged cardiomyocyte was also detected. This could be the reason why MIF levels in the early stage of STEMI patients were related to the final myocardial infarction area.

In addition to proinflammatory effects, MIF can also play a role in myocardial protection and its protective mechanism is mainly through activating the AMPK signaling pathway to upregulate glucose transport and utilization that provides for metabolic adaptation to ischemia or inhibiting the JNK signaling pathway to promote cell proliferation and inhibit apoptosis [20]. In our study, the cardiac protective role of MIF could be limited because of the myocardial damage caused by stress hyperglycemia or diabetes [9]. Elevated MIF appeared to exert an insignificant effect on cardiac protection.

We observed that MIF levels, in the early stage of the disease, were indicative of long-term cardiac function and prognosis in nondiabetes STEMI patients, possibly because of the involvement of MIF in the inflammatory response and the regulation of cardiac remodeling and fibrosis after myocardial infarction. Inflammatory response and fibrosis induced after myocardial infarction are important factors in determining the severity of ventricular remodeling and long-term cardiac function [21]. With the exacerbation of myocardial ischemia and infarction, MIF promotes the accumulation of macrophages and other inflammatory cells in damaged necrotic myocardium, upregulates an inflammatory response, and induces the production of other inflammatory factors, aggravating myocardial damage [10, 21–25]. In the ischemia/reperfusion model, White et al. and Liehn et al. observed that the collagen content in the infarcted area was significantly reduced one week after myocardial infarction by knocking out the mouse *MIF* gene or using MIF-neutralizing antibodies [23, 26]. There is evidence that MIF can also affect interstitial fibrosis in noninfarcted areas after myocardial infarction [27]. Myocardial biopsy of the heart of patients with nonischemic heart failure revealed that myocardial MIF content was positively correlated with the degree of cardiac fibrosis and was an independent risk factor for adverse cardiovascular events [28].

Makino et al. found that MIF levels were independent predictors of prognosis in patients with stable coronary heart disease with diabetes [29]. However, in the present study, in patients with acute STEMI and diabetes, the difference in the risk of adverse cardiovascular events between the high-level MIF group and the low-level group was not significant, possibly because of the small sample size, and thus, further analysis with larger sample size needs to be conducted in the future.

MIF is implicated in the pathogenesis of several immunoinflammatory and autoimmune diseases. Recently, the homologue of MIF, MIF-2 (also named D-dopachrome tautomerase), is attracting interest as an additional proinflammatory mediator in MS and experimental allergic encephalomyelitis [17, 30]. MIF-2, expressed in most tissues and by a variety of immune cells, circulates in the serum at similar concentrations as MIF and induces signaling cascades similar to MIF [20, 31, 32]. However, it remains to be demonstrated experimentally whether MIF-2 is activated in acute myocardial infarction in a similar manner as MIF. Currently, we do not have commercial detection methods to evaluate MIF-2 levels in human blood. Given the relationship between MIF and MIF-2, we could speculate that the MIF family, MIF and MIF-2, could be utilized to advance diagnosis or serve as a warning function but this is yet to be tested.

The data from this study not only suggest that MIF can be used as a biomarker in the clinical setting but also suggest that MIF could be a promising therapeutic target candidate in preventing cardiac dysfunction after acute myocardial infarction. Numerous experimental and clinical data support the regulatory role of MIF/MIF-2 in inflammatory response, which promotes the development of pharmacological strategies to inhibit the directional pathway of MIF/MIF-2 for therapeutic applications [20]. Novel chemical inhibitors of MIF and MIF-2 in myocardial infarction models remain to be tested.

There are limitations in the current study worth noting. First, this prospective cohort study had a small sample size; thus, it does not allow mechanistic interpretation of our findings. Second, this was a single-center study, limiting our subject pool. Third, cardiac infarct size affects MIF levels in STEMI patients [3]. Although we assessed the peak TnT value post-PCI to evaluate infarct size, more precise diagnostic methods, such as cardiac magnetic resonance examination, were not performed in these patients. Therefore, further large-scale investigations and careful comparisons are required to confirm the predictive ability of MIF in the long-term prognosis in patients with acute myocardial infarction complicated with diabetes.

## 5. Conclusions

In patients with acute myocardial infarction with or without diabetes, early blood MIF levels can reflect the extent of impaired cardiac function in the acute phase. In patients with acute myocardial infarction without diabetes, MIF levels can predict cardiac function and long-term prognosis at the 12-month follow-up. The mechanism may be associated with myocardial fibrosis related to stress and inflammation. Our study results suggest that MIF further affects patient survival with myocardial ischemia and myocardial fibrosis after myocardial infarction by participating in myocardial inflammation caused by hyperglycemia and diabetes, which may be related to the long-term prognosis of patients. Therefore, it is evident that MIF is a novel biomarker for heart disease and is worthy of further study in the future.

## Figures and Tables

**Table 1:** Baseline characteristics of the participants.

Characteristics	Diabetes STEMI group (*n* = 57)	Nondiabetes STEMI group (*n* = 147)	Control group (*n* = 65)
Age (mean ± SD) (years)	60.6 ± 11.5	58.6 ± 13.5	57.6 ± 11.2
Male, *n* (%)	44 (77.2)	124 (84.4)	51 (78.4)
BMI (mean ± SD) (kg/m^2^)	25.7 ± 2.9	25.7 ± 3.5	22.9 ± 5.5
eGFR (mean ± SD) (mL·min^−1^·kg^−1^)	83.5 ± 29.3^∗†^	92.3 ± 29.3	93.2 ± 26.6
Systolic pressure at presentation (mean ± SD) (mmHg)	132.2 ± 19.6	131.0 ± 18.2	125.1 ± 26.1
Diastolic pressure at presentation (mean ± SD) (mmHg)	76.8 ± 12.4	77.0 ± 12.7	72.1 ± 16.4
Heart rate at presentation (mean ± SD) (beats/min)	78.1 ± 17.8	76.2 ± 14.9	75.5 ± 17.3
Onset to visit time < 6 h, *n* (%)	47 (82.5)	112 (76.2)	—
Anterior myocardial infarction, *n* (%)	22 (38.6)	67 (45.6)	—
Killip grade ≥ II, *n* (%)	15 (26.3)^†^	19 (12.9)	—
LVEF ($\overset{¯}{x}\pm s$) (%)	53.3 ± 9.2	53.7 ± 8.6	—
Hypertension, *n* (%)	42 (73.7)^†^	62 (42.2)	—
Hyperlipidemia, *n* (%)	19 (33.3)	52 (35.4)	—
Active smoker, *n* (%)	37 (64.9)	106 (72.1)	—
White blood cell count (mean ± SD × 109/L)	10.4 ± 3.0^∗^	10.5 ± 3.2^∗^	6.59 ± 1.2
Peak value of CK-MB [*M*(Q25, Q75)] (U/L)	203 (141-300)	192 (149, 322)	—
Peak value of hs-TnT [*M*(Q25, Q75)] (ng/mL)	4.0 (2.3-6.3)	4.3 (2.4-6.2)	—
NT-pro-BNP [*M*(Q25, Q75)] (pg/mL)	1005 (438-2490)	992 (373, 1864)	—
hs-CPR [*M*(Q25, Q75)] (pg/mL)	6.0 (2.0-15.7)^∗^	6.9 (3.0-15.6)^∗^	0.9 (0.2-2.7)
LDL-C (mean ± SD) (mmol/L)	2.76 ± 0.78^∗^	2.92 ± 0.90^∗^	2.36 ± 0.74
Random blood glucose [*M*(Q25, Q75)] (mmol/L)	9.7 (5.8–12.4)	5.5 (4.9–7.1)	—
HbAlc (mean ± SD) (%)	7.1 ± 2.3^†^	6.0 ± 1.8	—

Data are presented either as mean ± SD, percentage, or median (25th percentile, 75th percentile). Categorical variables are indicated as percentage (%) of patients. ^∗^*P* < 0.05 compared to the control group; ^†^*P* < 0.05 compared to the nondiabetes STEMI group. BMI: body mass index; eGFR: estimated glomerular filtration rate; LVEF: left ventricular ejection fraction; CK-MB: isoenzyme of creatine kinase-muscle/brain; hs-TnT: high-sensitive troponin T; Nt-pro-BNP: N-terminal pro-b-type natriuretic peptide; hs-CPR: hypersensitive C-reactive protein; LDL-C: low-density lipoprotein cholesterol; HbAlc: glycosylated hemoglobin.

## Data Availability

The data used to support the findings of this study are available from the corresponding author upon request.
